# Omics-Based Insights into Flavor Development and Microbial Succession within Surface-Ripened Cheese

**DOI:** 10.1128/mSystems.00211-17

**Published:** 2018-01-30

**Authors:** A. S. Bertuzzi, A. M. Walsh, J. J. Sheehan, P. D. Cotter, F. Crispie, P. L. H. McSweeney, K. N. Kilcawley, M. C. Rea

**Affiliations:** a Teagasc Food Research Centre, Moorepark, Fermoy, County Cork, Ireland; b School of Food and Nutritional Science, University College Cork, Cork, Ireland; c School of Microbiology, University College Cork, Cork, Ireland; d APC Microbiome Institute, University College Cork, Cork, Ireland; University of California, San Diego

**Keywords:** dairy science, flavor, microbiology

## Abstract

In this study, a young Cheddar curd was used to produce two types of surface-ripened cheese, using two commercial smear-culture mixes of yeasts and bacteria. Whole-metagenome shotgun sequencing was used to screen the microbial population within the smear-culture mixes and on the cheese surface, with comparisons of microorganisms at both the species and the strain level. The use of two smear mixes resulted in the development of distinct microbiotas on the surfaces of the two test cheeses. In one case, most of the species inoculated on the cheese established themselves successfully on the surface during ripening, while in the other, some of the species inoculated were not detected during ripening and the most dominant bacterial species, *Glutamicibacter arilaitensis*, was not a constituent of the culture mix. Generally, yeast species, such as *Debaryomyces hansenii* and *Geotrichum candidum*, were dominant during the first stage of ripening but were overtaken by bacterial species, such as *Brevibacterium linens* and *G. arilaitensis*, in the later stages. Using correlation analysis, it was possible to associate individual microorganisms with volatile compounds detected by gas chromatography-mass spectrometry in the cheese surface. Specifically, *D. hansenii* correlated with the production of alcohols and carboxylic acids, *G. arilaitensis* with alcohols, carboxylic acids and ketones, and *B. linens* and *G. candidum* with sulfur compounds. In addition, metagenomic sequencing was used to analyze the metabolic potential of the microbial populations on the surfaces of the test cheeses, revealing a high relative abundance of metagenomic clusters associated with the modification of color, variation of pH, and flavor development.

**IMPORTANCE** Fermented foods, in particular, surface-ripened cheese, represent a model to explain the metabolic interactions which regulate microbial succession in complex environments. This study explains the role of individual species in a heterogeneous microbial environment, i.e., the exterior of surface-ripened cheese. Through whole-metagenome shotgun sequencing, it was possible to investigate the metabolic potential of the resident microorganisms and show how variations in the microbial populations influence important aspects of cheese ripening, especially flavor development. Overall, in addition to providing fundamental insights, this research has considerable industrial relevance relating to the production of fermented food with specific qualities.

## Abstract (precis)

Fermented foods, in particular, surface-ripened cheese, represent a model to explain the metabolic interactions which regulate microbial succession in complex environments. This study explains the role of individual species in a heterogeneous microbial environment, i.e., the exterior of surface-ripened cheese. Through whole-metagenome shotgun sequencing, it was possible to investigate the metabolic potential of the resident microorganisms and show how variations in the microbial populations influence important aspects of cheese ripening, especially flavor development. Overall, in addition to providing fundamental insights, this research has considerable industrial relevance relating to the production of fermented food with specific qualities.

## INTRODUCTION

Recent studies involving both metabolomic and metagenomic analyses have begun to address the relationship between the microbiota and biochemical pathways during the fermentation process (1–4). It is clear that in fermented food, the metabolic interactions which regulate the composition of the microbial population influence the taste, shelf life, and safety of the subsequent product (5). The ability to manipulate the microbiota of fermented food represents an important avenue for the food industry for developing new food products with precise characteristics.

Surface-ripened cheese (e.g., Münster, Tilsit, Livarot, Limburger, and Comté) is characterized by the growth of a heterogeneous microbiota on the cheese surface, with the consequent development of a strong flavor. The flavor and the appearance of these types of cheese are related to the metabolic activities of bacteria and yeasts, which comprise the smear consortium. Generally, the cheese is brined or surface salted, which also influences the growth of surface microbiota. In some traditional procedures, young cheese is smeared by transferring the smear from older cheese to a younger curd (old-young technique) (6, 7). However, today, commercial mixtures of smear bacteria and yeasts are more commonly used to produce a more standardized product.

So far, metagenomic sequencing represents a valid method to investigate the microbial population on the exterior of surface-ripened cheese (4, 8–10). In studies of complex microbial communities in fermented foods, such as kefir, the information gained through whole-metagenome shotgun sequencing allowed the variations of the microbial population and also the metabolic pathways involved in the fermentation process to be monitored (1).

The aim of the current study was to investigate, at both the species and the strain level, the succession of the microbial populations present on the rind of a surface-ripened cheese produced with young Cheddar cheese curd as a base, using two different commercial smear-culture mixes. Studies were performed over the course of 30 days of ripening to correlate volatile analysis with data generated through whole-metagenome shotgun sequencing in order to understand how microbial composition relates to flavor development. Moreover, metagenomic analysis allowed for the screening of metagenomic clusters during cheese ripening, showing the involvement of the surface microbiota in a variety of biochemical processes.

## RESULTS

### Microbial compositions of the smear-culture mixes.

**FIG 1:**
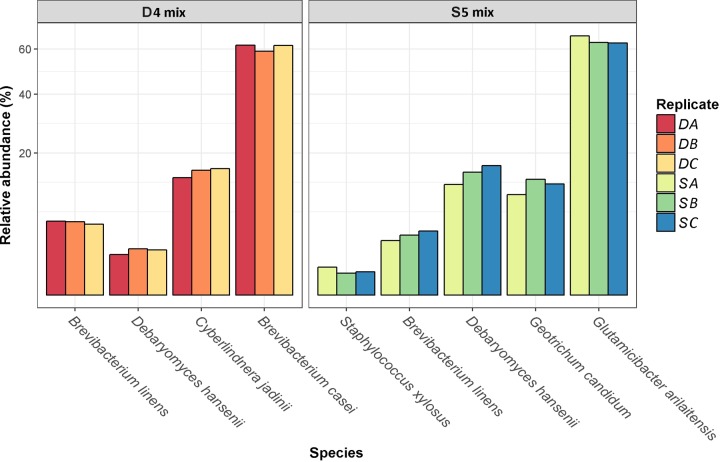
Relative abundances of the species (percentages) which were indicated as being present by the supplier within the smear-culture mixes D4 and S5 (results are from replicates of three analyses [DA, DB, DC and SA, SB, SC]).

Two smear-culture mixes, D4 and S5, were used for the cheese trials and contained, as outlined in the supplier specification sheet, *Brevibacterium linens*, *Debaryomyces hansenii*, *Cyberlindnera jadinii*, and *Brevibacterium casei* (for D4) or *Staphylococcus xylosus*, *B. linens*, *D. hansenii*, *Geotrichum candidum*, and *Glutamicibacter arilaitensis* (previously classified as *Arthrobacter arilaitensis*) (for D5). Using metagenomic analysis, performed with Kaiju (11), the relative abundances of the individual species within the mixes were determined (Fig. 1). Overall, Kaiju was able to assign 81.7% ± 1.5% of reads from the starter mix samples to the species level. The proportion of assigned reads for each starter mixture sample is presented in Fig. S1 in the supplemental material. *B. casei* (60.83%) and *C. jadinii* (15%) were the most abundant bacterial and yeast species in D4, while *B. linens* and *D. hansenii* were minor components in the smear-culture mix, with relative abundances of 5.25% and 1.92%, respectively (Fig. 1; Table S1). In the S5 mix, *G. arilaitensis* (64.25%), *D. hansenii* (14.56%), and *G. candidum* (11.83%) were the most abundant bacteria and yeasts; *S. xylosus* (0.59%) and *B. linens* (3.52%) were present at lower relative abundances. Other species, not specified by the suppliers, were identified at low relative abundances in the smear-culture mixes D4 and S5 and are reported in Table S1.

10.1128/mSystems.00211-17.1 FIG S1  Proportions of reads by Kaiju of samples of the smear-culture mixes. Download FIG S1, TIF file, 0.1 MB. Copyright © 2018 Bertuzzi et al. 2018 Bertuzzi et al. This content is distributed under the terms of the Creative Commons Attribution 4.0 International license.

10.1128/mSystems.00211-17.8 TABLE S1  Relative abundances (percentages) of the microbial species within the D4 and S5 mixes. Data are the means from 3 replicates. Species highlighted in bold were stated as present by the culture provider. Download TABLE S1, DOCX file, 0.02 MB. Copyright © 2018 Bertuzzi et al. 2018 Bertuzzi et al. This content is distributed under the terms of the Creative Commons Attribution 4.0 International license.

### Microbial compositions of the cheese surfaces.

Two test cheeses, D4 and S5, were prepared by smearing young Cheddar cheese curd with the two aforementioned commercial smear-culture mixes and ripened for 30 days at 15°C. Kaiju was used to determine the bacterial and yeast compositions of the cheese surfaces at days 0, 18, 24, and 30 for both the control cheese (unsmeared and ripened under vacuum) and the two test cheeses (11). Overall, Kaiju was able to assign 57.5% ± 8.3% of reads from the cheese samples to the species level. The proportions of assigned reads for each cheese sample are presented in Fig. S2. Compositional data of the cheese surface were analyzed by a one-way analysis of variance (ANOVA), designed with SAS 9.3, to determine the significant differences in the proportions of the individual species present over time (12). The metagenomic sequences of the bacteria used as starter cultures in the Cheddar cheese curd (*Lactococcus lactis* and *Streptococcus thermophilus*) and as smearing cultures (*B. linens*, *S. xylosus*, and *G. arilaitensis*) were compared at the strain level, using PanPhlAn, to determine the presence/absence of the inoculated bacterial strains on the cheeses throughout ripening (13).

10.1128/mSystems.00211-17.2 FIG S2  Proportions of reads by Kaiju (A) and SUPER-FOCUS (B) of the samples of the cheese surface. Download FIG S2, TIF file, 0.5 MB. Copyright © 2018 Bertuzzi et al. 2018 Bertuzzi et al. This content is distributed under the terms of the Creative Commons Attribution 4.0 International license.

**FIG 2:**
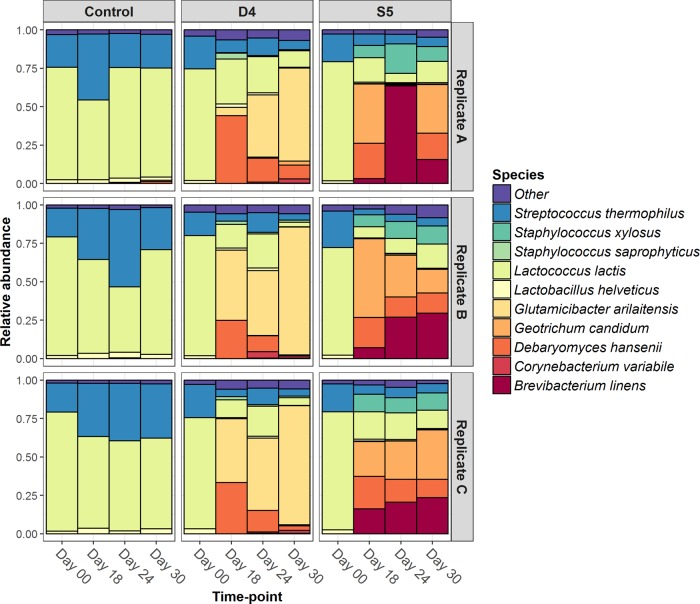
Relative abundances at the species level of the microbiotas on the surfaces of the control, D4, and S5 cheeses at days 0, 18, 24, and 30. Data shown are from the three replicate trials (A, B, and C).

**FIG 3:**
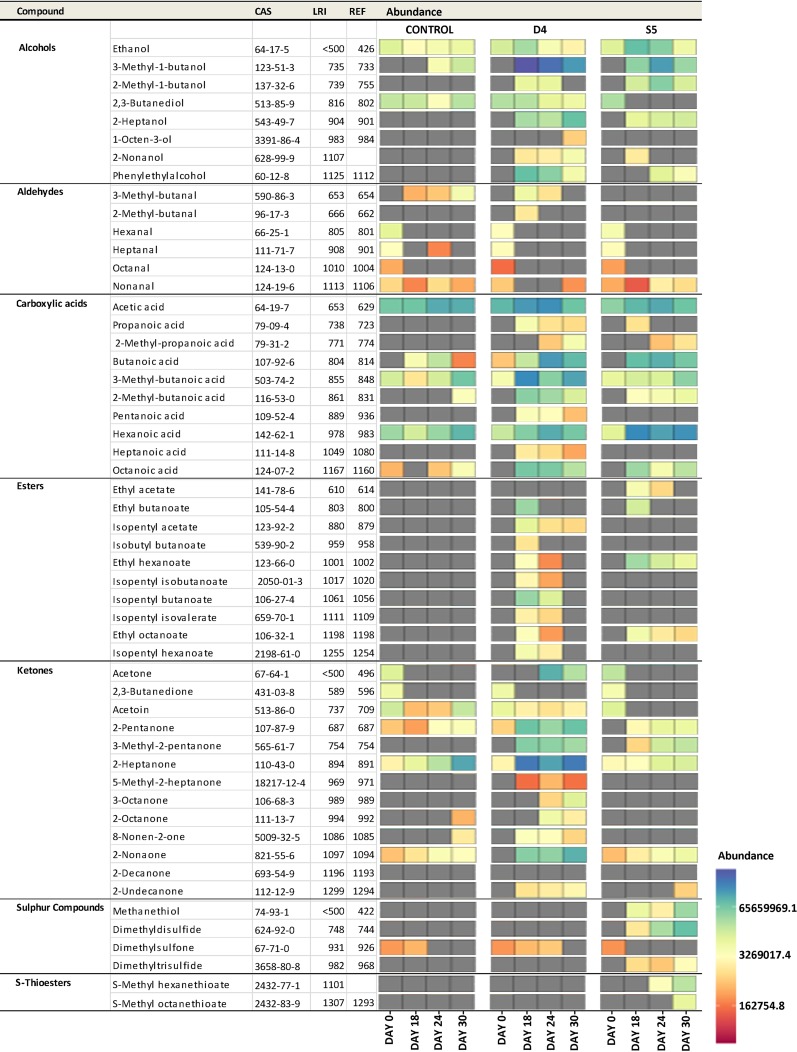
Volatile compounds detected in cheese by GC-MS and faceted heat map showing the variation of volatile compounds between the cheeses at days 0, 18, 24, and 30. The gray tiles indicate when the volatile compounds were not detected. The linear retention index (LRI) was calculated and compared with the reference linear retention index (REF) to confirm the identification. Values are the means of results from three replicates.

As expected, lactic acid bacteria dominated the surfaces of all samples at day 0, and their relative abundances on the surface of the control cheese did not significantly change throughout the 30 days of ripening (Fig. 2). *L. lactis* and *S. thermophilus* were identified in all samples analyzed (D4, S5, and control) (Fig. 3). *L. lactis* was the dominant species in the control, constituting 75.85% of the initial population at day 0 and decreasing to 65.99% at day 30. *S. thermophilus* increased from 19.65% at day 0 to 28.21% at day 30, while the relative abundance of *Lactobacillus* (*Lb*.)* helveticus* was low throughout the ripening period (2.12% at day 0 and 2.72% at day 30) (Table S2). However, over the course of 30 days of ripening, the smearing processes clearly influenced the microbial populations of the cheese surfaces of both test cheeses, D4 and S5, causing a significant reduction in the relative abundances of *Lb. helveticus* (*P <* 0.03) and *L. lactis* (*P <* 0.0001). From day 0 to day 18, the population on the surface of D4 changed from predominately lactic acid bacteria to *Debaryomyces hansenii* and *Glutamicibacter arilaitensis* (Fig. 2). Subsequently, over the course of ripening, the relative abundance of *D. hansenii* significantly decreased (*P <* 0.0001) from 34.12% at day 18 to 4.14% at day 30 (Table S2). In parallel, the relative abundance of *G. arilaitensis* significantly increased (*P <* 0.0001) from 30.9% at day 18 to become the dominant population on the cheese surface (73.75%) at day 30 (Table S2). Using PanPhlAn, it was determined that the strain of *G. arilaitensis* detected on the cheese surface of D4 was different from the *G. arilaitensis* strain used in the smear-culture mix inoculated onto the surface of S5, confirming that the growth of this strain on D4 did not result from cross contamination of the two cheeses during inoculation or ripening (Fig. S3 and S4). However, the *G. arilaitensis* strain detected on D4 did appear to be more closely related to the strain on the control cheeses (Fig. S4). The secondary microbial population (individually between 1% and 3% of the population) of the D4 surface was composed of species not included in the initial smear-culture mix and included *Arthrobacter* sp., *Corynebacterium variabile*, *Debaryomyces fabryi*, *G. candidum*, *Staphylococcus equorum*, and *Staphylococcus saprophyticus* (Table S2). In addition, some species present in the initial smear-culture mix (*C. jadinii* and *B. casei*) were not detected during ripening, while the inoculated *B. linens* strain was detected at only at a very low relative abundance on the cheese surface of D4 throughout ripening (Table S2).

10.1128/mSystems.00211-17.3 FIG S3  Heat maps showing the relatedness of strains detected in cheese samples to reference strains, as determined by PanPhlAn, based on the presence/absence of pangenome gene families in their respective genomes. The term “reference” refers to the reference genome sequences of strains present in the NCBI databases and highly similar to the genome sequences of the strains identified in the smear mixes or on the cheese surface. Download FIG S3, TIF file, 2.1 MB. Copyright © 2018 Bertuzzi et al. 2018 Bertuzzi et al. This content is distributed under the terms of the Creative Commons Attribution 4.0 International license.

10.1128/mSystems.00211-17.4 FIG S4  Principal-component analysis (PCA) plot of the profiles of the strains determined by PanPhlAn. Download FIG S4, TIF file, 0.2 MB. Copyright © 2018 Bertuzzi et al. 2018 Bertuzzi et al. This content is distributed under the terms of the Creative Commons Attribution 4.0 International license.

10.1128/mSystems.00211-17.9 TABLE S2  Relative abundances of the microbial species on the surfaces of the control, D4, and S5 cheeses at days 0, 18, 24, and 30. Data are means from 3 replicates. Download TABLE S2, DOCX file, 0.02 MB. Copyright © 2018 Bertuzzi et al. 2018 Bertuzzi et al. This content is distributed under the terms of the Creative Commons Attribution 4.0 International license.

By comparison, the microbiota was more diverse in cheese S5 (Fig. 2; Table S2). On the cheese surface of S5, the relative abundances of the lactobacilli decreased, while that of *B. linens* increased significantly (*P <* 0.004) from day 18 to day 24, reaching 37.05% before decreasing, but not significantly, to 22.84% at day 30 (Table S2). The strain detected was confirmed by PanPhlAn to be that inoculated within the S5 mix (Fig. S3 and S4). The yeasts *D. hansenii* and *G. candidum* (components of the S5 mix) were the most abundant populations on the cheese surface at day 18, comprising 21.2% and 37.54% of the microbiota, respectively, but their relative abundances significantly decreased (*P <* 0.04) by day 24 to 9.57% and 17.6%, respectively, without showing further significant reductions at day 30 (Table S2). *S. xylosus*, did not correspond to the strain present in the S5 mix (Fig. S3 and S4) and was detected at 9.08% at day 18 but did not change significantly throughout the ripening period (Table S2). In addition, a secondary microbial population, comprising *D. fabryi* (detected in the S5 mix) (Table S1) and *Psychrobacter* sp. (not detected in the S5 mix) (Table S1), developed at low relative abundance (1 to 2%) on the surface of cheese S5 (Table S2) over the course of the ripening period. However, some inoculated species were either not detected (*S. equorum*) at any stage throughout ripening or detected at a very low relative abundance (*G. arilaitensis*, ~0.44%) on the cheese surface during ripening (Table S2).

### Volatile compounds present on the cheese surface.

Headspace solid-phase microextraction (HS-SPME) gas chromatography-mass spectrometry (GC-MS) was used to analyze the development of volatile compounds at days 0, 18, 24, and 30 of ripening for both the control and test cheeses. In total, 53 volatile compounds that potentially contributed to the flavor development were detected on the cheese surfaces. These compounds are predicted to arise from a variety of substrates and consisted of 8 alcohols, 6 aldehydes, 10 carboxylic acids, 10 esters, 13 ketones, 2 *S-*thioesters, and 4 sulfur compounds (i.e., a total of 53 compounds) (Fig. 3). As expected, given the microbial diversity on the surface, there was a greater variety and intensity of volatile compounds detected than on the control cheese, on which only 23 of the aforementioned 53 compounds were detected (Fig. 3). In all cheeses, the levels of all volatile compounds detected increased throughout the ripening period, apart from those of 2,3-butanediol, hexanal, heptanal, octanal, nonanal, 2,3-butanedione, and dimethylsulfone (Fig. 3).

### Correlations between microbial taxa and volatile compounds.

**FIG 4:**
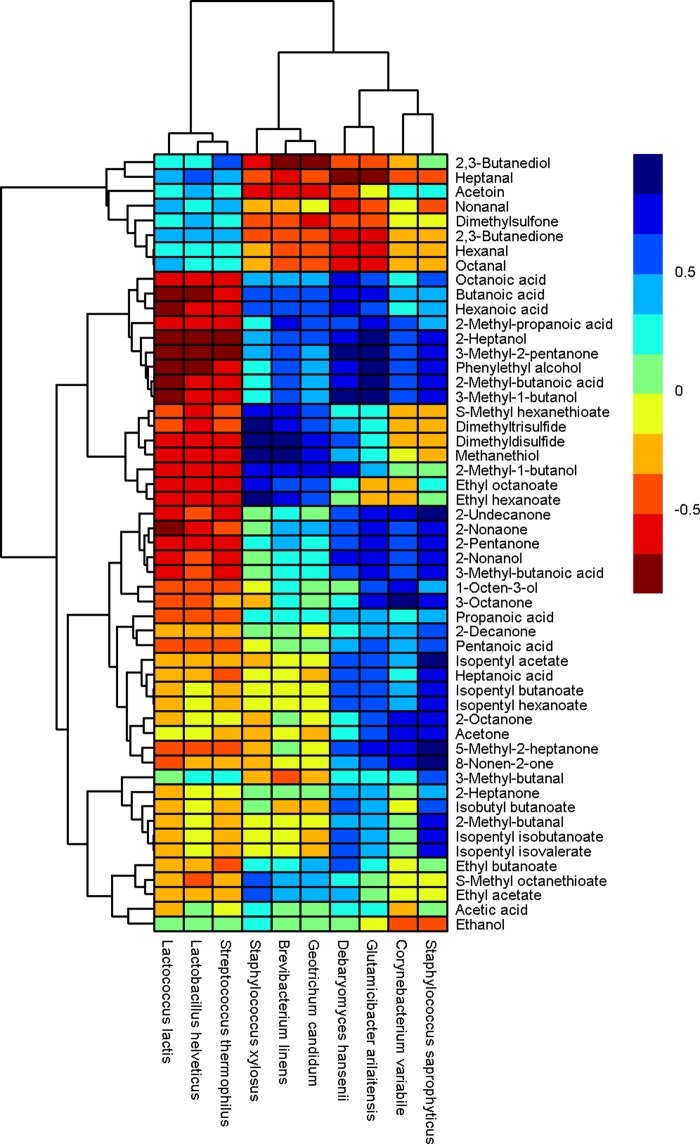
Hierarchically clustered map showing the correlation between the relative abundances of the microbial species and the levels of volatile compounds detected on the cheese surface. Clustering was performed by using the hclust function in R. The color of each tile of the heat map indicates the level of correlation for a given species-compound combination, as indicated by the color key.

The correlation analysis between the relative abundances of microbial species and the abundances of volatile compounds detected on the cheese surface was performed using the Spearman correlation test, as described previously by Walsh et al. (1). From the results of the metagenomic analysis (performed with Kaiju) and the volatile analysis, it was possible to associate both yeasts and bacteria, at the species level, with specific volatile compounds. Figure 4 demonstrates the degrees of correlation between the volatile compounds and the organisms detected.

There was a strong correlation between *B. linens* and *G. candidum* and sulfur compounds and 2-methyl-1-butanol. *S. xylosus* was correlated with sulfur compounds, 2-methyl-1-butanol, and some ethyl esters; *Corynebacterium variablile* was correlated with ketones. *D. hansenii* was correlated with acids and alcohols, *G. arilaitensis* was correlated with ketones, alcohols, and acids, and *S. saprophyticus* was correlated with ketones, esters, acids, and alcohols (Fig. 4; Table 1).

**TABLE 1:** List of strong positive correlations^a^ between the levels of volatile compounds and the relative abundances of species on the cheese surface

Correlation species and compound	Potential precursor	*R* value
*Debaryomyces hansenii*		
2-Methyl butanoic acid	Isoleucine	0.81
3-Methyl-1-butanol	Leucine	0.85
Octanoic acid	Lipolysis	0.76
Hexanoic acid	Lipolysis	0.81
2-Heptanol	2-Heptanone (fatty acid oxidation)	0.8

*Glutamicibacter arilaitensis*		
2-Methyl butanoic acid	Isoleucine	0.9
3-Methyl-1-butanol	Leucine	0.86
3-Methyl butanoic acid	Leucine	0.77
Phenylethyl alcohol	Phenylalanine	0.83
3-Methyl-2-pentanone	Fatty acid oxidation	0.89
2-Undecanone	Fatty acid oxidation	0.82
5-Methyl-2-heptanone	Fatty acid oxidation	0.78
2-Pentanone	Fatty acid oxidation	0.77
2-Nonaone	Fatty acid oxidation	0.76
2-Heptanol	2-Heptanone (fatty acid oxidation)	0.86
	
*Geotrichum candidum*		
2-Methyl-1-butanol	Isoleucine	0.76
Methanethiol	Methionine	0.76
Dimethyldisulfide	Methanethiol	0.79

*Brevibacterium linens*		
2-Methyl-1-butanol	Isoleucine	0.81
Methanethiol	Methionine	0.82
Dimethyldisulfide	Methanethiol	0.85
Dimethyltrisulfide	Methanethiol	0.77
	
*Staphylococcus xylosus*		
2-Methyl-1-butanol	Isoleucine	0.77
Methanethiol	Methionine	0.84
Dimethyldisulfide	Methanethiol	0.95
Dimethyltrisulfide	Methanethiol	0.86
Methylthio hexanoate	Methanethiol + hexanoic acid	0.78
Ethyl hexanoate	Ethanol + hexanoic acid	0.85
Ethyl octanoate	Ethanol + octanoic acid	0.77

*Staphylococcus saprophyticus*		
2-Methyl-butanoic acid	Isoleucine	0.76
3-Methyl-1-butanol	Leucine	0.77
Heptanoic acid	Lipolysis	0.76
5-Methyl-2-heptanone	Fatty acid oxidation	0.98
2-Undecanone	Fatty acid oxidation	0.88
8-Nonen-2-one	Fatty acid oxidation	0.87
3-Methyl-2-pentanone	Fatty acid oxidation	0.77
2-Nonanol	2-Nonaone (fatty acid oxidation)	0.78
Isopentyl acetate	3-Methyl-1-butanol + acetic acid	0.87
Isopentyl butanoate	3-Methyl-1-butanol + butanoic acid	0.8
Isopentyl hexanoate	3-Methyl-1-butanol + hexanoic acid	0.8

*Corynebacterium variabile*		
3-Octanone	Fatty acid oxidation	0.99
2-Octanone	Fatty acid oxidation	0.78
5-Methyl-2-heptanone	Fatty acid oxidation	0.77

a Correlations for which the *P* value was <0.001 (corrected for multiple comparisons using the Bonferroni method) and the *R* value was >0.75.

### Gene content of cheese surface microbiota.

**FIG 5:**
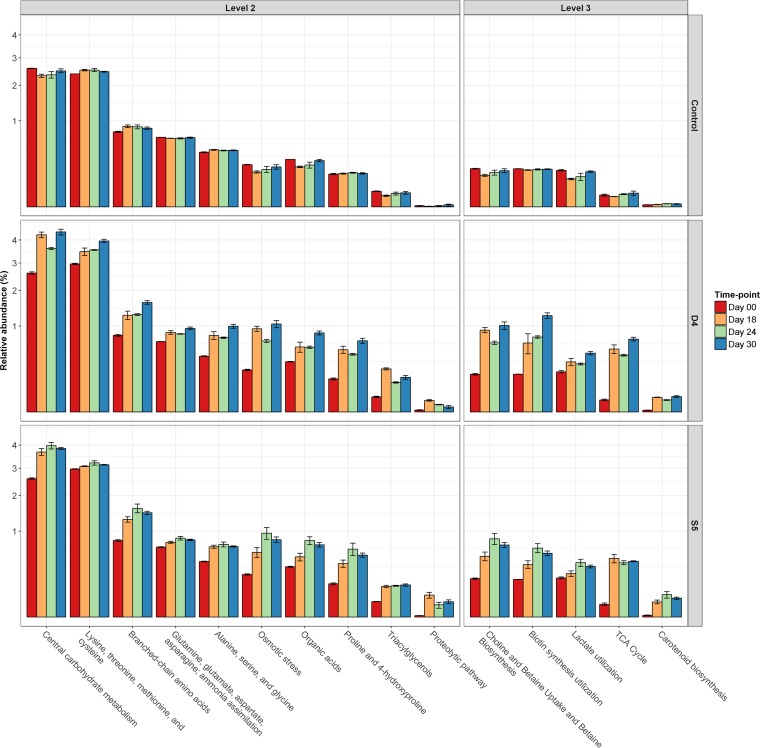
Averages and standard errors from the three replicate trials of the relative abundances of significantly different (*P <* 0.05) metagenomic clusters detected with SUPER-FOCUS at days 0 (red), 18 (orange), 24 (green), and 30 (blue) for the surfaces of the control, D4, and S5 cheeses.

Using SUPER-FOCUS (https://edwards.sdsu.edu/SUPERFOCUS) (14), whole-metagenome shotgun sequencing was used to characterize the functional potential of the whole microbial community on the cheese surfaces at different stages of ripening. Overall, SUPER-FOCUS was able to assign 62.5% ± 10.9% of reads from the cheese samples to a function. The proportions of assigned reads for each cheese sample are presented in Fig. S2. The functional clusters analyzed were initially organized into three different levels, in relation to the specificity of the metabolic pathways. Pathway data were analyzed to determine the significant differences of the individual metabolic clusters by ANOVA, using SAS 9.3 (12), with the selection of 16 specific functional clusters with relative abundances significantly higher (*P <* 0.05) on the cheese surfaces of S5 and D4 than on that of the control (Fig. 5).

### Color and pH variation.

pH and color analyses were performed on the three cheese types, and the resultant data were examined using a split-plot test, designed with SAS 9.3 (12). A significant interactive effect (*P <* 0.0001) between smear treatments and ripening time was observed for pH. At days 18, 24, and 30, the pH was significantly higher (*P <* 0.0001) on the surfaces of S5 and D4 than on that of the control. In addition, the pH was significantly higher (*P <* 0.0001) on the surface of S5 than on that of D4 from day 18 onwards (Fig. S5).

10.1128/mSystems.00211-17.5 FIG S5  Changes in the pHs of the surfaces of the control (circles), D4 (squares), and S5 (triangles) cheeses. Data show the means and standard deviations of results from three replicate trials. Download FIG S5, TIF file, 0.04 MB. Copyright © 2018 Bertuzzi et al. 2018 Bertuzzi et al. This content is distributed under the terms of the Creative Commons Attribution 4.0 International license.

A significant interactive effect (*P <* 0.0001) between time and smear treatments was observed for L*, a*, and b* values. The L* value measures the visual lightness (as values increase from 0 to 100), the a* value measures the redness to greenness (positive to negative values, respectively), and the b* value measures the yellowness to blueness (positive to negative values, respectively). At days 18, 24, and 30, the a* value was significantly higher (*P <* 0.0001) for the surfaces of S5 and D4 than for the surface of the control. At day 30, the a* value was also significantly higher (*P <* 0.02) on the surface of D4 than on that of S5 (Fig. S6).

10.1128/mSystems.00211-17.6 FIG S6  Color development on the surfaces of the control (circles), D4 (squares), and S5 (triangles) cheeses. Data show the means and standard deviations of results from three replicate trials. Download FIG S6, TIF file, 0.1 MB. Copyright © 2018 Bertuzzi et al. 2018 Bertuzzi et al. This content is distributed under the terms of the Creative Commons Attribution 4.0 International license.

### FAA and FFA analyses.

Free amino acid (FAA) and free fatty acid (FFA) analyses were performed on the three cheese types, and the experimental results were examined by one-way ANOVA, using SAS 9.3 (12). The concentrations of total FAAs on the surfaces of S5 (15,158 ± 1,683 µg ⋅ mg^−1^) and D4 (11,914 ± 1,769 µg ⋅ mg^−1^) were significantly higher (*P <* 0.05) than those on the control surface (6,605 ± 819 µg ⋅ mg^−1^). In addition, the concentrations of some individual FAAs, such as tyrosine, proline, and histidine, were significantly higher (*P <* 0.05) on the surface of S5 than on the surfaces of D4 and the control (Fig. S7).

10.1128/mSystems.00211-17.7 FIG S7  Free amino acid (A) and free fatty acid (B) concentrations (micrograms per milligram) on the surfaces of the control (red), D4 (green), and S5 (yellow) cheeses at day 30. Data show the means of results from three replicate trials. The significant differences (*P <* 0.05) are indicated with a, b, and c. Download FIG S7, TIF file, 0.1 MB. Copyright © 2018 Bertuzzi et al. 2018 Bertuzzi et al. This content is distributed under the terms of the Creative Commons Attribution 4.0 International license.

The concentrations of total FFAs on the surfaces of S5 (22,069 ± 3,875 µg ⋅ mg^−1^) and D4 (26,562 ± 2,606 µg ⋅ mg^−1^) were significantly higher (*P <* 0.05) than on the surface of the control (1,336 ± 70 µg ⋅ mg^−1^). The concentrations of some individual FFAs, such as C_4:0_, C_8:0_, C_10:0_, C_12:0_, C_14:0_, and C_18:0_, were significantly higher (*P <* 0.05) on the surface of D4 than on that of S5 or the control (Fig. S7).

## DISCUSSION

In this study, the use of whole-metagenome shotgun sequencing facilitated the study, at the species and the strain level, of microbial succession among smear microorganisms (both bacteria and yeasts) on cheese surfaces and facilitated the analysis of the metabolic potential of the whole microbial community at different stages of ripening. Volatile flavor compounds were analyzed over time, using HS-SPME GC-MS, and correlated with the microbial species that developed during ripening.

Cheddar cheese curd <24 h postmanufacture was inoculated with two different smear-culture mixes and incubated at 15°C for 30 days. Unsmeared Cheddar cheese curd, vacuum packed to prevent the growth of spoilage molds on the cheese surface, was used as a control. This model was chosen to investigate microbial succession and flavor development, as it had been shown in a previous study that yeasts and bacteria establish themselves satisfactorily on the surface of young Cheddar cheese curd, producing cheese with modified flavor and appearance (15).

On the cheese surfaces of S5 and D4, a very heterogeneous microbial consortium developed during ripening, triggering an array of biochemical processes. Yeasts are considered to be responsible for the deacidification of the cheese surface (observed on S5 and D4) (Fig. S5) by the degradation of lactate (to CO_2_ and H_2_O) (16, 17), as well as for the formation of alkaline metabolites (from metabolism of FAAs) (18) and the secretion of growth factors (vitamins and amino acids) that support the growth of bacteria (17, 19). As expected, in parallel with the growth of the yeasts, the relative abundances of the metagenomic clusters related to lactate utilization and the biosynthesis and uptake of biotin were greater for the cheese surfaces of D4 and S5 than for that of the control (Fig. 5). During ripening, the surfaces of D4 and S5 were washed with a 5% salt solution, causing hyperosmotic stress on the microbial population of the cheese surface (20). This correlated with higher relative abundances of the metagenomic clusters related to osmotic-stress resistance and the metabolism of choline and betaine (osmoprotectants) (21) for the washed cheeses than for the unwashed control (Fig. 5).

The development of a red/orange color on the surface is an important characteristic of many smear-ripened cheeses. This color development is usually derived through the metabolism of carotenoids (22, 23), and correspondingly higher relative abundances of metagenomic clusters involved in carotenoid biosynthesis were observed on the surfaces of the cheeses S5 and D4 than on that of the control (Fig. 5).

Surface-ripened cheeses are also characterized by a strong flavor, which is driven by the biochemical metabolism of the microbial consortium that develops on the cheese surface over time. These are associated with proteolytic and lipolytic pathways, driving the increase in the levels of FAAs and FFAs. These pathways, together with lactose and citrate metabolism, are considered to be responsible for the main precursors of flavor compounds in cheese. In the current study, the relative abundances of the metagenomic clusters associated with the proteolytic pathway and the metabolism of triacylglycerols were higher for D4 and S5 than for the control, which was consistent with FAA- and FFA-related data (Fig. S7). During ripening, the relative abundances of metagenomic clusters directly related to the formation of volatile compounds, such as carbohydrates, organic acids (including FFAs), and FAAs (except aromatic amino acids), and of clusters indirectly related to the formation of volatile compounds, such as those used in the tricarboxylic acid (TCA) cycle (important for α-ketoglutarate production), were significantly higher (*P <* 0.05) for the surfaces of both the D4 and S5 cheeses than for that of the control cheese (Fig. 5). Correspondingly, numerous volatile compounds (alcohols, aldehydes, carboxylic acids, ketones, sulfur compounds, esters, and *S-*thioesters) (Fig. 3) were produced on the surfaces of cheeses S5 and/or D4, conferring an intense flavor to them.

During ripening, on the cheese surfaces of S5 and D4, a microbial succession involving various inoculated, and indeed some noninoculated, microorganisms was apparent. Consistently with other studies, specific smear strains, added as adjunct cultures to the milk or to the exterior of surface-ripened cheese during manufacture, have not been detected at the end of ripening (24–28). In this study, the species detected on the cheese surface by metagenomic analysis did not fully correspond with the components of the smear-culture mixes. Different contaminant populations developed on the surfaces of both test cheeses, especially on that of D4, probably due to the different interactions and competition between the cultures of the two mixes (Fig. 2; Table S2).

*D. hansenii* was part of the inoculum used for both S5’s and D4’s surface. *D. hansenii* is a component of the surface microbiota of many surface-ripened cheeses and is very tolerant to high-salt and low-pH conditions (16, 29). Presumably due to these characteristics, *D. hansenii* was present at a high relative abundance in both test cheeses, mainly in the early stage of ripening (at day 18), and then decreased gradually in the later stages (days 24 and 30) (Table S2). Volatile compounds significantly (*P <* 0.001) associated with *D. hansenii* were mainly alcohols and carboxylic acids (Fig. 4; Table 1). The biosynthesis of branched-chain alcohols and carboxylic acids from FAA metabolism and the biosynthesis of medium-to-long carboxylic acids from FFA metabolism are processes attributed mainly to yeast and mold metabolism, including that of *D. hansenii* (30–35).

On cheese D4, the relative reduction of *D. hansenii* with time corresponded to an increase in the number of Gram-positive bacteria. *G. arilaitensis*, a component of S5’s mix, did not grow on the cheese surface of S5 and, though it was not inoculated as part of the culture mix, was the dominant bacterium on the surface of D4 (Fig. 2; Table S2). Through the use of PanPhlAn, which uses metagenomic data to achieve strain-level microbial profiling resolution, we have demonstrated that the *G. arilaitensis* strain present on D4 was not the same strain as inoculated onto S5 (Fig. S3 and S4). The inability of the inoculated *G. arilaitensis* strain to grow on the S5 cheese is most likely due to the different interactions within the microbiota on the cheese surface. Other studies on the microbial composition of the surface of Limburger cheese observed that *G. arilaitensis* behaved in a similar manner, showing high relative abundance when it was coinoculated only with *D. hansenii* but showing low relative abundance when combined with both *D. hansenii* and *G. candidum* (17). That *G. arilaitensis* contributes to cheese flavor has been shown previously in model cheese media (36) (producing alcohols and especially ketones) and in the current study, where it was significantly (*P <* 0.001) associated with 3-methyl-1-butanol and phenylethyl alcohol, branched carboxylic acids (from FAA metabolism), 2-heptanol, and ketones (from FFA metabolism) (Fig. 4; Table 1). In addition, a genomic study showed numerous genes encoding protein degradation and fatty acid oxidation in *G. arilaitensis* (37).

On the cheese surface of S5, *G. candidum* was coinoculated with *D. hansenii* and established itself to become the most abundant yeast population by day 18. The successful cohabitation of *G. candidum* and *D. hansenii* may be explained by the fact that they do not compete for energy sources in the same way in cheese. *D. hansenii* uses lactate or the limited amount of lactose present in the cheese postmanufacture (0.8 to 1%), while *G. candidum* preferentially uses only lactate (21, 38). During ripening, sulfur compounds were significantly (*P <* 0.001) associated with *G. candidum* (Fig. 4; Table 1), which is in agreement with other studies which have shown that *G. candidum* is able to catabolize methionine in a one-step degradation, with the biosynthesis of sulfur compounds (34, 39, 40).

The production of sulfur compounds is an important characteristic of many surface-ripened cheese, and *B. linens* is considered one of the main species responsible for the development of the strong flavor of many surface-ripened cheeses through the biosynthesis of sulfur compounds derived from methanethiol. In this study, *B. linens* was present at relatively low abundances in the original culture mixes (5.26% and 3.53% for D4 and S5, respectively) (Table S1). However, although it was detected at a very low relative abundance on the cheese surface of D4, it was one the most dominant bacteria detected on S5 (37.05% at day 24) (Table S2). While this may be due to interstrain differences, it is most likely due to the different interactions within the microbiotas of S5 and D4. Studies have shown that *B. linens* does not always establish itself on the cheese surface during ripening, even if it is present in the initial culture mix (25–27, 41, 42). However, in previous studies, *G. candidum* has been shown to stimulate the growth of *B. linens* in coculture (43), suggesting the hypothesis that in S5, *G. candidum*, present at high relative abundance, might have produced growth factors that supported the growth of *B. linens* but that in D4, it was out-competed by *G. arilaitensis*, which established itself very quickly on the surface of S5 and made up 75% of the microbiota at the end of ripening. *B. linens* was significantly (*P <* 0.001) associated with methanethiol and its derivatives (dimethyldisulfide and dimethyltrisulfide) (Fig. 4; Table 1), which likely originated from the one-step degradation of methionine (30, 36, 44, 45).

Other species, while present at lower relative abundances on the cheese surfaces of S5 and D4, were also responsible for the biosynthesis of some volatile compounds. A strain of *S. xylosus* different from the one within the smear-culture mix of S5 (Fig. S4) was detected during ripening only at 10.83 to 13.36% of its relative abundance on the cheese surface of S5 (Table S2). This is most likely due to competition for nutrients within the microbiota, as suggested by Mounier et al. (38). Members of the genus *Staphylococcus* can establish themselves on surface-ripened cheese in the early stages of ripening but are regularly overtaken by other bacteria at the later stages (26, 46, 47).

In this study, specific species detected in low relative abundances in S5, such as *S. xylosus* (9.08 to 13.36%), and in D4, such as *S. saprophyticus* (1.06 to 2.69%) and *C. variable* (2.04 to 2.08%) (Table S2), were significantly (*P <* 0.001) associated with a range of flavor compounds important in surface-ripened cheese (Fig. 4; Table 1), and interestingly, while *S. xylosus* has previously been shown to produce sulfur compounds only in fermented meat (48, 49), in this study, it was correlated with specific sulfur compounds in cheese. These data suggest that some smear bacteria, though present at relatively low abundances in cheese, are likely contributors to the release of FFAs and to their degradation due to their esterase activity and, hence, that they contribute to the aroma and flavor in the final cheese product (50, 51).

In the study reported here, whole-metagenome shotgun sequencing was employed as a novel method for the analysis of a fermented product with a complex microbiota. Metagenomic analysis was an efficient tool to understand the variations of the microbial population of the cheese surface over time and the related metabolic potential. Moreover, the association between the volatile compounds and the species represents a novel system for studying flavor development in cheese. In conclusion, the approach used in this study enabled us to determine the microbial succession during ripening and also to begin to unravel the contributions of the various components of the surface microbiota when present within a complex microbial environment. The method proposed in this study can be adopted in industry to control the microbiotas of fermented food, resulting in the production of food products with specific flavor characteristics.

## MATERIALS AND METHODS

### Smearing of cheese blocks.

A block of commercial Cheddar cheese <24 h after manufacture was aseptically cut into smaller blocks (~8 by 6.5 by 30 cm) and washed with smearing solutions, as described in our previous study (15). Two commercial smear-culture mixes comprising *G. candidum*, *D. hansenii*, *B. linens*, *G. arilaitensis*, and *S. xylosus* (S5 mix) (Sacco, Cadorago, Italy) and *D. hansenii*, *C. jadinii*, *B. casei*, and *B. linens* (D4 mix) (DuPont Danisco, Beaminster, Dorset, United Kingdom) were used to inoculate the surfaces of the cheese curds. The blocks of cheese were washed with the smearing solutions and placed in sterile racks inside sterile plastic bags (Südpack Verpackungen, Ochsenhausen, Germany), as previously described (15). The cheese was ripened for 30 days at 15°C, with a relative humidity of ~97%. At days 7, 10, and 15 of ripening, the cheese blocks were brushed with a sterile sponge that had been soaked in a sterile brine solution (5% NaCl) to uniformly spread the smear microbiota on the cheese surface. As a control, unsmeared cheese blocks were vacuum packed in sterile bags and incubated at 15°C, as with the test cheeses.

### Sampling cheese.

Three replicate cheese trials were performed at different times during Cheddar cheese making season. All data presented are the results of the analysis performed on samples taken from the cheese surface (at a depth of ~0.5 cm). All analyses were performed in triplicate.

### pH measurement.

The pH level was measured on days 0, 18, 24, and 30 using a standard pH meter (MP220; Mettler-Toledo, Schwerzenbach, Switzerland) (52). The data were analyzed by one-way analysis of variance (ANOVA) using SAS 9.3 (12).

### Determination of color.

At days 0, 18, 24, and 30 of ripening, the color was measured on the cheese surface at room temperature, using a Minolta CR-300 colorimeter (Minolta Camera, Osaka, Japan). The instrument was calibrated on white tile, and the color of the cheese surface was measured using L*, a*, and b* values. The L* value measures the visual lightness (as values increase from 0 to 100), the a* value measures the redness to greenness (positive to negative values, respectively), and the b* value measures the yellowness to blueness (positive to negative values, respectively).

### Total DNA extraction from the cheese surface.

The total DNA was extracted from the smear culture mixes and the cheese samples using the PowerSoil DNA isolation kit as described in the manufacturer’s protocol (Cambio, Cambridge, United Kingdom). For the DNA extraction from the cheese surface, at days 0, 18, 24, and 30, a pretreatment step was included as follows. Samples were removed from different parts of the cheese block and pooled to give a representative sample of 5 g. The cheese was placed in a stomacher bag with 50 ml of 2% trisodium citrate and homogenized using a masticator mixer (IUL SA, Barcelona, Spain) for 5 min.

Fifteen milliliters of the smear-culture mix, or the cheese solution, was placed into sterile Falcon tubes and centrifuged for 30 min at 4,500 × *g*. After centrifugation, the supernatant was discarded and the pellet was placed in a 2-ml Eppendorf tube. The pellet was washed several times with sterile phosphate-buffered saline (PBS) by centrifuging it at 14,500 × *g* for 1 min, until the supernatant was completely clear. The pellet was then added to PowerBead tubes (Cambio, Cambridge, United Kingdom) provided with the kit as described in the protocol and homogenized by shaking on the TissueLyser II (Qiagen, West Sussex, United Kingdom) at 20 Hz for 10 min. The DNA was then purified according to the protocol of the standard PowerSoil DNA isolation kit (Cambio, Cambridge, United Kingdom).

Total DNA was initially qualified and quantified by gel electrophoresis and the NanoDrop 1000 (Bio-Sciences, Dublin, Ireland) before more-accurate quantification with the Qubit high-sensitivity DNA assay (Bio-Sciences, Dublin, Ireland).

### Whole-metagenome shotgun sequencing.

Whole-metagenome shotgun libraries were prepared in accordance with the Nextera XT DNA library preparation guide from Illumina (53). Libraries for the starter mixture samples were sequenced on the Illumina MiSeq with a 2× 300-bp cycle v3 kit. Libraries for the cheese samples were sequenced on the Illumina NextSeq 500 with a v2 NextSeq 500/550 high-output reagent kit (300 cycles). All sequencing was done in the Teagasc sequencing facility in accordance with standard Illumina sequencing protocols.

### Bioinformatic analysis.

Raw whole-metagenome shotgun sequencing reads were processed on the basis of quality and quantity using a combination of Picard tools (https://github.com/broadinstitute/picard) and SAMtools (54). Processing of raw sequence data produced a total of 3,214,480 ± 841,719 filtered reads for samples sequenced on the MiSeq and 19,210,475 ± 12,478,696 filtered reads for samples sequenced on the NextSeq. The metagenomic binning tool Kaiju (11) was used to determine the species-level microbial compositions of samples. The NCBI nonredundant protein database (55) was used with Kaiju. PanPhlAn (13) was used for strain-level analysis of species of interest. PanPhlAn works by aligning sequencing reads against a species pangenome database, built from reference genomes, to identify the gene families present in strains from metagenomic samples. The reference genomes included for each pangenome database are outlined in Table S3. SUPER-FOCUS (14) was used to characterize the microbial metabolic potential of samples. SUPER-FOCUS measures the abundances of subsystems, or groups of proteins with shared functionality, by aligning sequencing reads against a reduced SEED (56) database.

10.1128/mSystems.00211-17.10 TABLE S3  Reference genomes used to generate the pangenomes for PanPhlAn analysis. Download TABLE S3, DOCX file, 0.02 MB. Copyright © 2018 Bertuzzi et al. 2018 Bertuzzi et al. This content is distributed under the terms of the Creative Commons Attribution 4.0 International license.

### Free amino acid analysis.

FAA analysis was performed at the end of the ripening (day 30) on the soluble N extracts using a JEOL JLC-500V AA analyzer fitted with a JEOL Na^+^ high-performance cation-exchange column (JEOL, Garden City, Herts, United Kingdom) (57). The chromatographic analyses were conducted at pH 2.2. Results are expressed as micrograms per milligram of cheese.

### Free fatty acid analysis.

FFA extractions were performed at the end of the ripening (day 30) according to the method outlined by De Jong and Badings (58). The FFA extracts were derivatized as methyl esters as described by Mannion et al. (59). Fatty acid methyl ester extracts were analyzed using a Varian CP3800 gas chromatograph (Aquilant, Dublin, Ireland) with a CP84000 autosampler and flame ionization detector and a Varian, Inc., 1079 injector (Aquilant, Dublin, Ireland). Results are expressed as micrograms per milligram of cheese.

### Volatile analysis.

The volatile compounds were analyzed at days 0, 18, 24, and 30. The surface of the cheese was removed, wrapped in foil, and stored vacuum packed at −20°C until analysis. Before analysis, the samples were defrosted and grated, and 4 g of the cheese surface was used. Analysis was carried out as outlined by Bertuzzi et al. (15).

### Statistical analysis.

Statistical analysis was done with SAS 9.3 (12) and R-3.2.2 (60). The R packages ggplot2 and pheatmap were used for data visualization. The vegan package was used to calculate the Bray-Curtis dissimilarity between samples, while the Hmisc package was used for correlation analysis.

### Accession number(s).

Sequencing reads have been deposited in the European Nucleotide Archive under the project accession number PRJEB15423.
