# Supplementary figures and images for: Omics-Based Insights into Flavor Development and Microbial Succession within Surface-Ripened Cheese
Source: mSystems. 2018 Jan 30;3(1):e00211-17. doi: 10.1128/mSystems.00211-17 (PMC5790873; doi:10.1128/mSystems.00211-17)

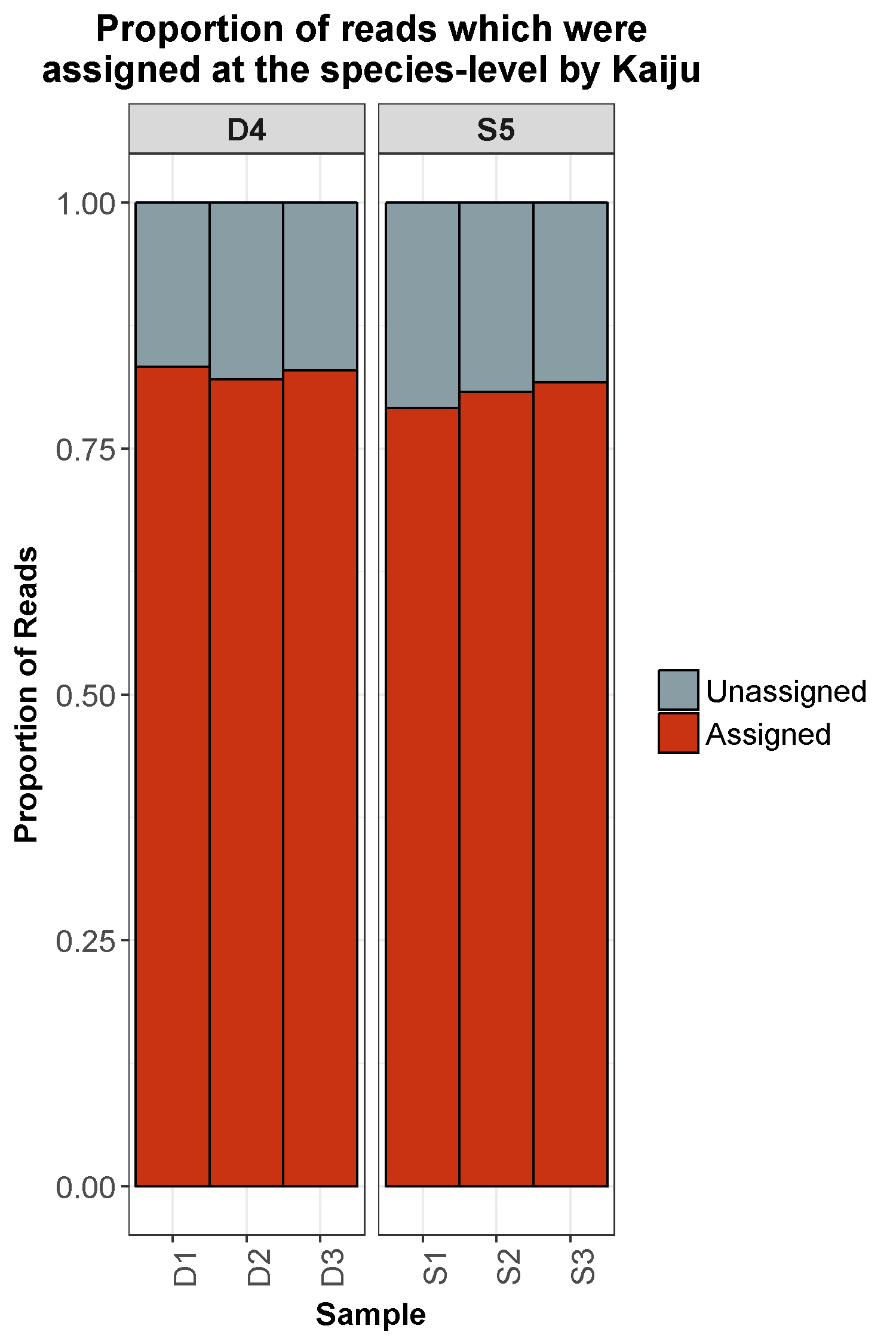

Supplement: FIG S1 [file sys001182167sf1.tif]

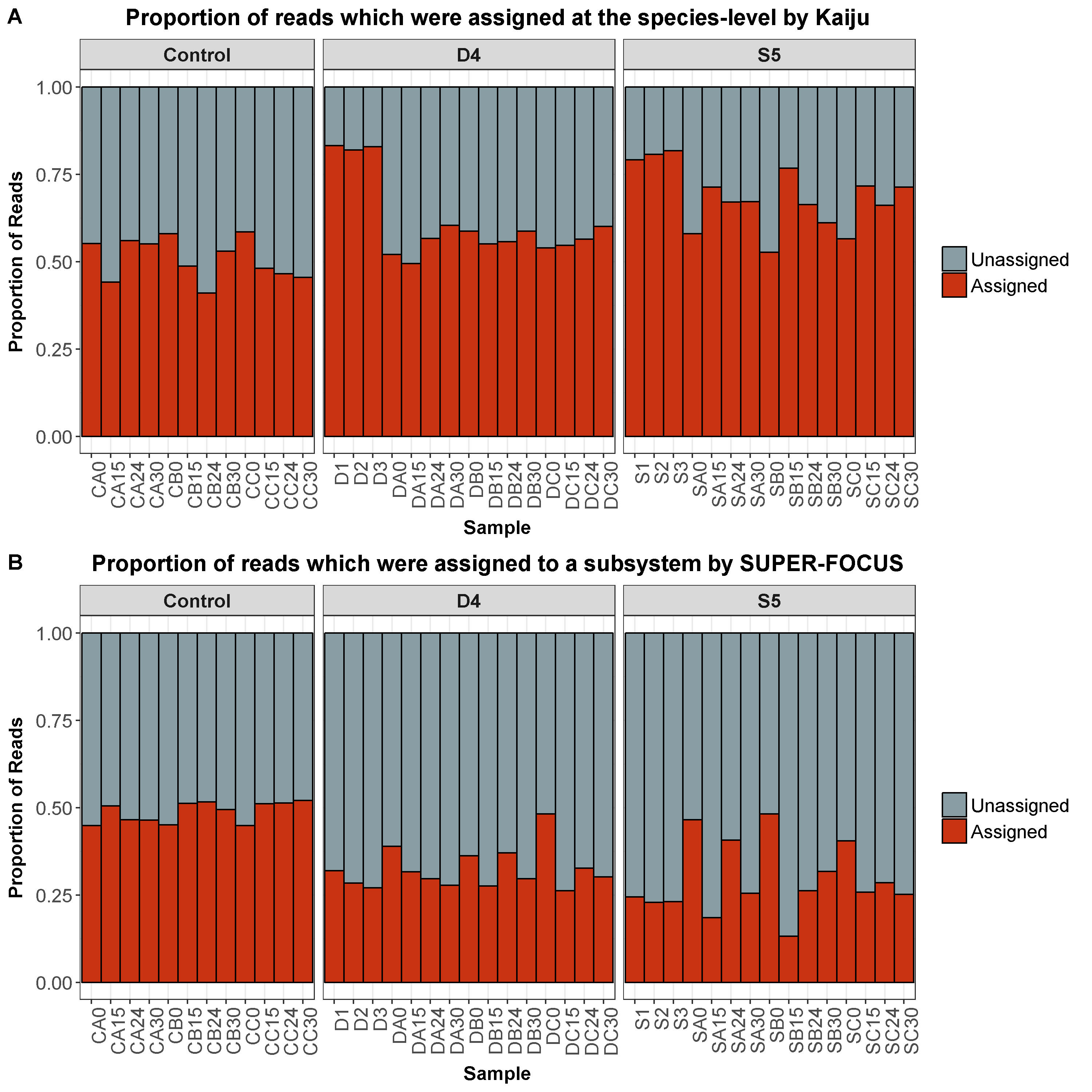

Supplement: FIG S2 [file sys001182167sf2.tif]

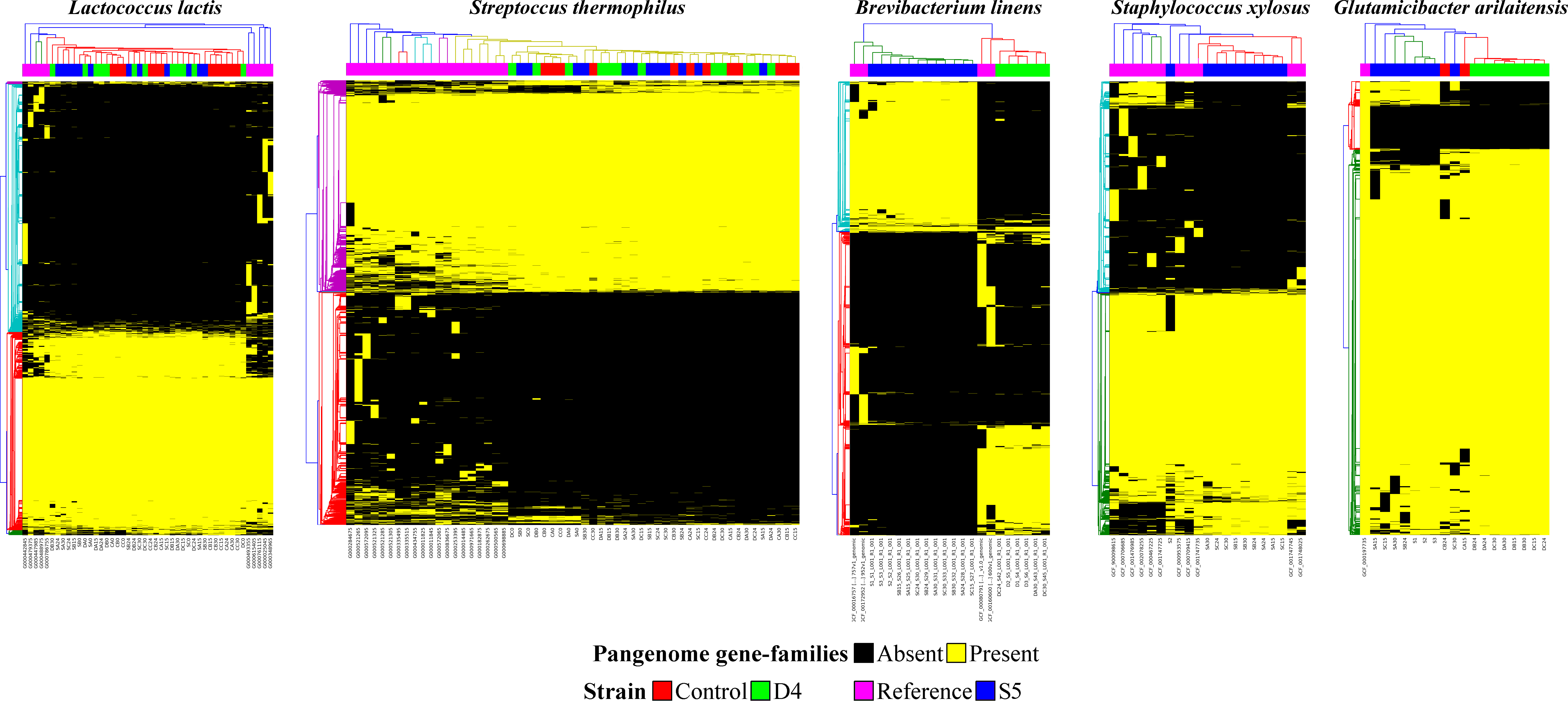

Supplement: FIG S3 [file sys001182167sf3.tif]

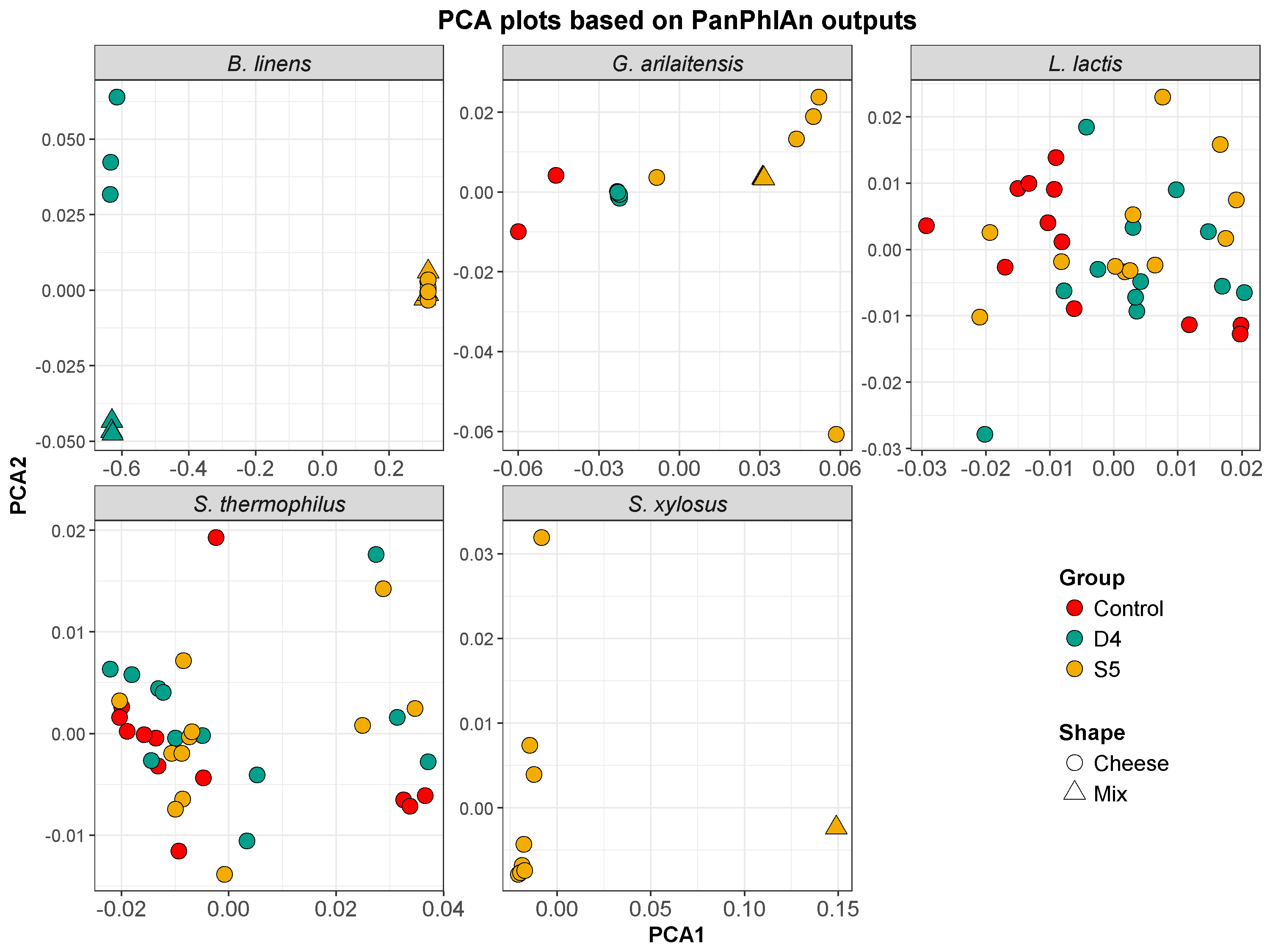

Supplement: FIG S4 [file sys001182167sf4.tif]

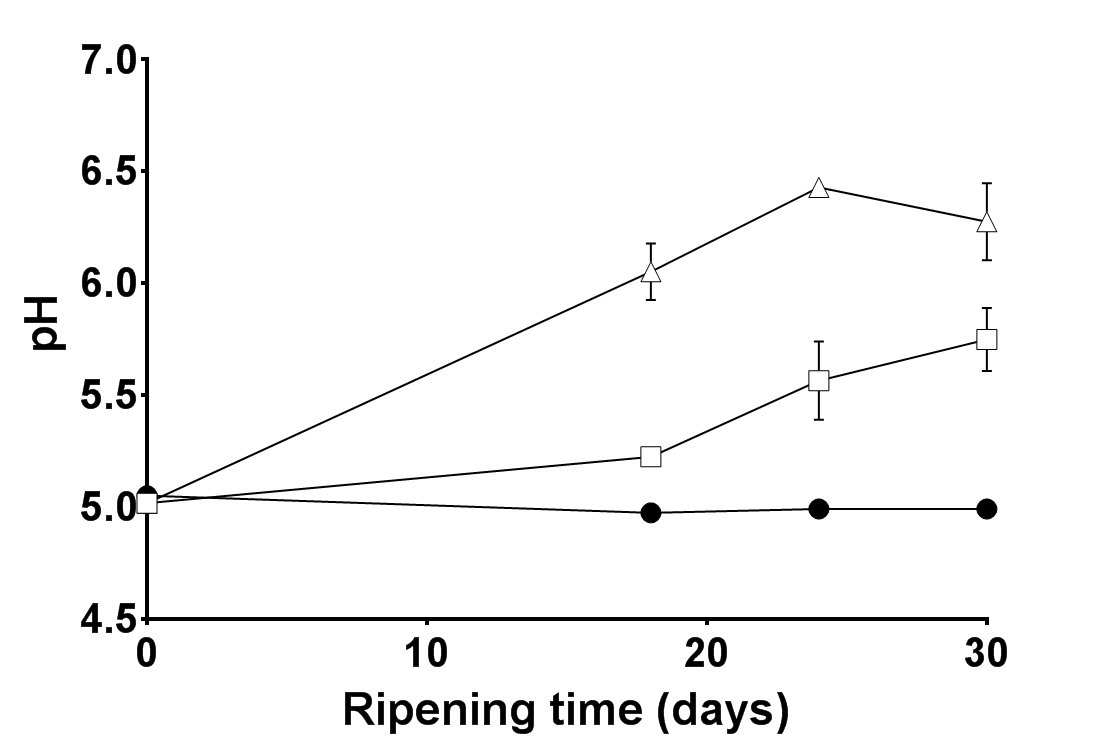

Supplement: FIG S5 [file sys001182167sf5.tif]

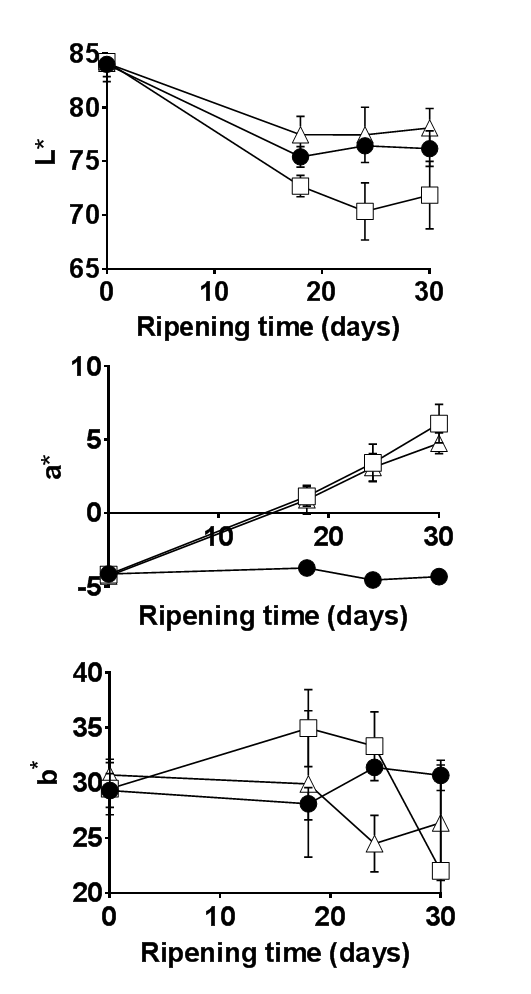

Supplement: FIG S6 [file sys001182167sf6.tif]

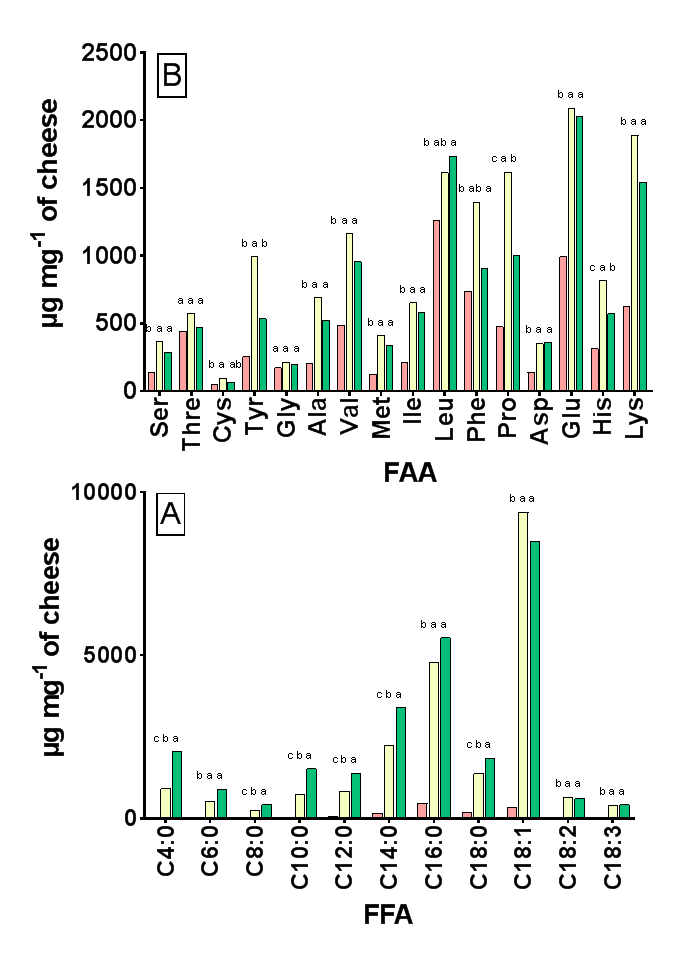

Supplement: FIG S7 [file sys001182167sf7.tif]
