# Supplementary material for: Omics-Based Insights into Flavor Development and Microbial Succession within Surface-Ripened Cheese
Source: mSystems. 2018 Jan 30;3(1):e00211-17. doi: 10.1128/mSystems.00211-17 (PMC5790873; doi:10.1128/mSystems.00211-17)
Supplement: TABLE S1 [file sys001182167st1.docx]

| **Species in the smear-culture mixes (%)** | **D4 mix** | **S5 mix** |
| --- | --- | --- |
| ***Brevibacterium casei*** | **61.09** | -^nd^ |
| ***Brevibacterium linens*** | **5.26** | **3.53** |
| ***Glutamicibacter arilaitensis*** | **-** | **64.03** |
| ***Staphylococcus xylosus*** | **-** | **0.57** |
| ***Cyberlindnera jadinii*** | **14.84** | - |
| ***Debaryomyces hansenii*** | **1.88** | **14.66** |
| ***Geotrichum candidum*** | **-** | **12.12** |
| *Brevibacterium sp. VCM10* | 12.82 |  |
| *Brevibacterium siliguriense* | 1.41 | - |
| *Brevibacterium epidermidis* | 1.10 | - |
| *Brevibacterium sandarakinum* | 0.59 | - |
| *Arthrobacter sp. NIO-1057* | - | 1.27 |
| *Debaryomyces fabryi* | - | 1.07 |
| *Arthrobacter sp. W1* | - | 0.38 |
| *Glutamicibacter mysorens* | - | 0.24 |
| *Arthrobacter sp. EpRS66* | - | 0.16 |
| *Paeniglutamicibacter antarcticus* | - | 0.14 |
| *Others* | 1.00 | 1.81 |

^nd^Not detected
