# Supplementary material for: Omics-Based Insights into Flavor Development and Microbial Succession within Surface-Ripened Cheese
Source: mSystems. 2018 Jan 30;3(1):e00211-17. doi: 10.1128/mSystems.00211-17 (PMC5790873; doi:10.1128/mSystems.00211-17)
Supplement: TABLE S2 [file sys001182167st2.docx]

|  | **Control** | | | | **D4** | | | | **S5** | | | |
| --- | --- | --- | --- | --- | --- | --- | --- | --- | --- | --- | --- | --- |
| **Species** | **0** | **18** | **24** | **30** | **0*** | **18** | **24** | **30** | **0*** | **18** | **24** | **30** |
| *Lactococcus lactis* | 75.85 | 57.58 | 57.76 | 65.99 | 74.35 | 18.7 | 21.72 | 6.18 | 74.74 | 13.59 | 11.02 | 13.94 |
| *Streptococcus thermophilus* | 19.65 | 36.93 | 36.53 | 28.21 | 19.4 | 6.03 | 11.66 | 4.98 | 20.04 | 5.79 | 5.91 | 5.82 |
| *Glutamicibacter arilaitensis* | -^nd^ | - | 0.24 | - | - | 30.9 | 43.27 | 73.75 | - | 0.26 | 0.44 | 0.42 |
| *Debaryomyces hansenii* | - | - | 0.07 | 0.45 | - | 34.12 | 13.29 | 4.14 | - | 21.2 | 9.57 | 14.09 |
| *Geotrichum candidum* | - | - | 0.08 | 0.28 | - | - | 0.31 | 1.11 | - | 37.54 | 17.6 | 26.44 |
| *Brevibacterium linens* | - | - | - | - | - | - | 0.17 | 0.26 | - | 8.84 | 37.05 | 22.84 |
| *Staphylococcus xylosus* | - | - | - | - | - | 0.11 | - | - | - | 9.08 | 13.36 | 10.83 |
| *Lactobacillus helveticus* | 2.12 | 3.1 | 2.78 | 2.72 | 2.38 | 1.39 | 1.41 | 0.34 | 2.19 | 0.86 | 0.48 | 0.46 |
| *Acinetobacter baumannii* | 0.82 | 0.12 | 0.17 | 0.37 | 0.63 | - | - | - | 0.95 | - | - | 0.15 |
| *Streptococcus pneumoniae* | 0.56 | 0.79 | 0.74 | 0.66 | 0.95 | - | 0.29 | 0.06 | 0.74 | 0.12 | 0.06 | - |
| *Streptococcus salivarius* | 0.5 | 0.93 | 0.93 | 0.71 | 0.52 | - | 0.3 | - | 0.5 | 0.11 | 0.06 | 0.05 |
| *Arthrobacter sp. NIO-1057* | - | - | - | - | - | 0.57 | 0.72 | 1.29 | - | - | - | - |
| *Staphylococcus equorum* | - | - | - | - | - | 1.32 | 0.43 | 0.45 | - | - | - | - |
| *Staphylococcus saprophyticus* | - | - | - | - | - | 2.69 | 0.93 | 1.06 | - | - | - | - |
| *Penicillium camemberti* | - | - | - | - | - | 0.37 | 0.4 | 0.63 | - | - | - | - |
| *Corynebacterium variabile* | - | - | - | - | - | - | 2.04 | 2.08 | - | - | - | - |
| *Debaryomyces fabryi* | - | - | - | - | - | 1.47 | 0.56 | 0.13 | - | 1.64 | 0.71 | 1.09 |
| *Psychrobacter sp. P11F6* | - | - | - | - | - | - | - | - | - | - | 0.4 | 0.5 |
| *Psychrobacter glacincola* | - | - | - | - | - | - | - | - | - | - | 0.83 | 1.13 |
| *Psychrobacter sp. JCM 18903* | - | - | - | - | - | - | - | - | - | - | 0.52 | 0.65 |
| *Stenotrophomonas maltophilia* | - | - | 0.2 | 0.37 | - | - | - | - | - | 0.17 | - | 0.18 |
| *Brevibacterium sandarakinum* | - | - | - | - | - | - | - | - | - | 0.2 | 0.94 | 0.58 |
| *Anaplasma phagocytophilum* | 0.22 | - | - | - | 0.74 | - | - | - | 0.45 | - | - | - |
| *Other* | 0.27 | 0.54 | 0.49 | 0.24 | 1.04 | 2.33 | 2.5 | 3.56 | 0.39 | 0.59 | 1.03 | 0.86 |

*Cheese D4 and S5 at day 0, before the smearing process

^nd^Not detected
