# Supplementary material for: Omics-Based Insights into Flavor Development and Microbial Succession within Surface-Ripened Cheese
Source: mSystems. 2018 Jan 30;3(1):e00211-17. doi: 10.1128/mSystems.00211-17 (PMC5790873; doi:10.1128/mSystems.00211-17)
Supplement: TABLE S3 [file sys001182167st3.docx]

| **Pangenome Database** | **Reference Strain (RefSeq Assembly Accession)** |
| --- | --- |
| *Brevibacterium linens* | GCF_000167575 |
|  | GCF_000807915 |
|  | GCF_001606005 |
|  | GCF_001729525 |
| *Glutamicibacter arilaitensis* | GCF_000197735 |
|  | GCF_000238915 |
|  | GCF_001302565 |
|  | GCF_002189495 |
| *Lactococcus lactis* | GCF_000014545 |
|  | GCF_000025045 |
|  | GCF_000143205 |
|  | GCF_000312685 |
|  | GCF_000348965 |
|  | GCF_000442845 |
|  | GCF_000447825 |
|  | GCF_000447845 |
|  | GCF_000447885 |
|  | GCF_000447985 |
|  | GCF_000468955 |
|  | GCF_000479375 |
|  | GCF_000488975 |
|  | GCF_000493355 |
|  | GCF_000534815 |
|  | GCF_000615405 |
|  | GCF_000731635 |
|  | GCF_000761115 |
|  | GCF_000786755 |
| *Streptococcus thermophilus* | GCF_000011825 |
|  | GCF_000011845 |
|  | GCF_000014485 |
|  | GCF_000182875 |
|  | GCF_000253395 |
|  | GCF_000262675 |
|  | GCF_000284675 |
|  | GCF_000335495 |
|  | GCF_000335515 |
|  | GCF_000434755 |
|  | GCF_000500565 |
|  | GCF_000521265 |
|  | GCF_000521285 |
|  | GCF_000521305 |
|  | GCF_000521325 |
|  | GCF_000572065 |
|  | GCF_000572095 |
|  | GCF_000698885 |
|  | GCF_000836675 |
|  | GCF_000971665 |
|  | GCF_001068405 |
|  | GCF_001071365 |
|  | GCF_001073445 |
| *Staphylococcus xylosus* | GCF_000338275 |
|  | GCF_000467225 |
|  | GCF_000706685 |
|  | GCF_000709415 |
|  | GCF_000815285 |
|  | GCF_000953575 |
|  | GCF_001476985 |
|  | GCF_001747725 |
|  | GCF_001747735 |
|  | GCF_001747745 |
|  | GCF_001748025 |
|  | GCF_001748045 |
|  | GCF_002078255 |
|  | GCF_900098615 |
